# Overcoming Resistance Mechanisms to Immune Checkpoint Inhibitors: Leveraging the Anti-Tumor Immune Response

**DOI:** 10.3390/curroncol31010001

**Published:** 2023-12-19

**Authors:** Courtney H. Coschi, Rosalyn A. Juergens

**Affiliations:** 1 Department of Oncology, McMaster University, 699 Concession Street, Hamilton, ON L8V 5C2, Canada; coschic@hhsc.ca; 2 Escarpment Cancer Research Institute, McMaster University, Hamilton, ON L8V 5C2, Canada

**Keywords:** immune checkpoint inhibitor, resistance, T cells, anti-tumor immune response, lung cancer

## Abstract

As far back as 3000 years ago, the immune system was observed to play a role in mediating tumor regression. Since then, many strategies have been developed to leverage the anti-tumor immune response. However, while many patients respond to ICIs up front some do not, and many of those that do eventually experience tumor progression. Currently, there are several predictive biomarkers of the immune checkpoint inhibitor response; however, no one test appears to be universally predictive and their application varies by disease site. There are many ways in which cancer cells develop primary or acquired resistance to immune checkpoint inhibitors. Efforts to reverse resistance include ways to combat T cell exhaustion, reprogram the tumor microenvironment, increase the availability of tumor neo-antigens, target alternative immune checkpoints, restore a normal/healthy patient gut microbiome, oncolytic viruses and tumor vaccines. The most studied and most promising methods include combining ICIs with therapies targeting alternative immune checkpoints and restoring a normal/healthy patient gut microbiome. This review will discuss T cell-mediated immunity, how this is leveraged by modern immunotherapy to treat cancer and mechanisms of immune checkpoint inhibitor resistance, while highlighting strategies to overcome primary and secondary resistance mechanisms.

## 1. Introduction

As far back as 3000 years ago, reports about the regression of tumors following infection and subsequent activation of the immune system have been documented [1,2]. Over time, several different physicians including Galen, Fehleisen and Busch independently described the regression of tumors after an episode of erysipelas in some patients [1,3]. It was not until 1891, though, that activation of the immune system was deliberately used to treat cancer. William Bradley Coley, who created Coley’s Toxins (mixtures of live and inactivated Streptococcus pyogenes and Serratia marcescens), injected this mixture directly into patients’ tumors and observed the responses. Coley reported success in a variety of malignancies for over 1000 patients and in 1908 biochemist Paul Ehrlich confirmed Coley’s observations, reporting that tumors could be spontaneously suppressed by actions of the immune system [2,4,5,6,7]. In 1909, Erlich subsequently proposed that cancerous cells arise in our bodies at a high frequency, but that our immune system must play a key role in ridding our bodies of them most of the time [8]. Concerns about infecting cancer patients with pathogenic bacteria, as well as failure to further the theory of immunotherapy to treat cancer in human trials, led to the rise and preference of surgery and radiotherapy to treat cancer in the early 1900’s.

It was not until the 1940’s that interest in the immune system as it related to cancer immunotherapy increased. Over the next few decades, interferon was discovered, the first cancer vaccine was trialed, the first bone marrow transplant to treat childhood leukemia took place, the Bacille Calmette-Guerin (BCG) vaccine was used to treat bladder cancer, and the characterization of different types of immune cells including T cells and natural killer (NK) cells occurred [9,10,11,12,13,14,15]. In 1957, Thomas and Burnet proposed that lymphocytes may act as immune-surveillance sentinels to identify and eliminate somatic cells with mutations that transformed them into cancer cells, and this was subsequently proven by Schreiber, Dunn and Old in the late 1990’s and early 2000’s [16,17]. The first immune checkpoint molecule, cytotoxic T-lymphocyte antigen 4 (CTLA-4), was discovered by Brunet et al. in 1987 however, its role as an inhibitor of immune activation/response was only confirmed in 1995 by Allison et al. [18,19,20]. Envisioning this as an important anti-cancer tool, the first immune checkpoint inhibitor (ipilimumab) was developed against CTLA-4 in 1996 and subsequently approved by the FDA in 2011 for treatment in advanced melanoma [20,21]. Approximately 20% of patients that participated in the first ipilimumab clinical trials remain alive without evidence of recurrence [21]. Following the success of ipilimumab, several other immune checkpoint inhibitors (ICIs) have been developed and approved for the treatment of various malignancies including, but not limited to avelumab, atezolizumab, durvalumab, nivolumab and pembrolizumab, targeting programmed death 1 (PD-1) and programmed death ligand 1 (PD-L1), which also act as immune checkpoints to downregulate immune response/activation against antigens.

While these ICIs have shown great success in improving survival for patients with certain tumor types including melanoma, lung and renal cell carcinoma, they have been met with less success in others including pancreatic and prostate cancers and glioblastoma. Efforts to develop predictive biomarkers of the ICI response include (i) the development of tests that score the degree of immune system engagement in the tumor microenvironment (TME) such as the tumor proportion score (TPS) which characterizes expression of PD-L1 on tumor cells, and the combined proportion score (CPS), which additionally looks at PD-L1 expression on immune cells (lymphocytes and macrophages); (ii) assessment of the mutation burden in the cancer cell genome by providing a tumor mutation burden (TMB) expressed as the number of mutations per megabase; and (iii) characterizing the level of cancer cell genomic instability as assessed by observing microsatellite instability (MSI) and reporting microsatellite status as MSI-high, MSI-low or microsatellite stable (MSS) [22]. However, no one test appears to be universally predictive and their application varies by disease site (ex. TPS in lung cancer, CPS in esophageal and breast cancers and MSI status in colorectal cancers).

Furthermore, while many patients respond to ICIs up front some do not, and many of those that do eventually experience tumor progression. This is due to either primary or secondary resistance to ICIs by cancer cells. The goal of this review is to briefly cover T cell-mediated immunity and how this is leveraged by modern immunotherapy to treat cancer, and mechanisms of tumor resistance to immunotherapies. The remainder of the review will focus on ways to therapeutically overcome primary and secondary resistance mechanisms.

## 2. T Cell-Mediated Immunity

**Figure 1:**
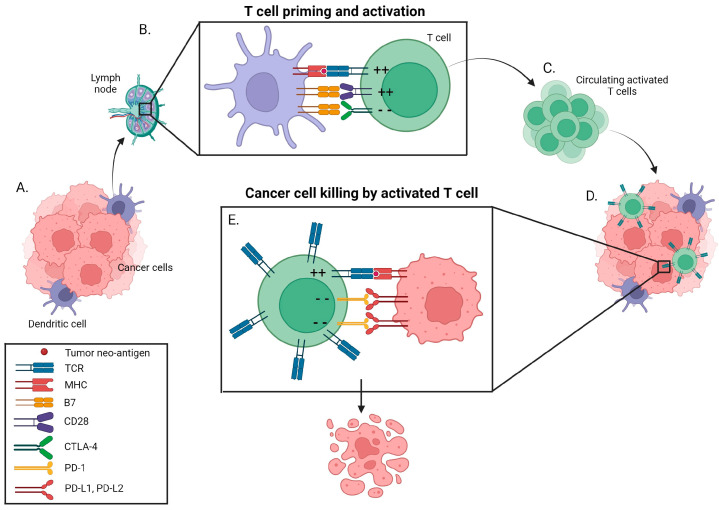
A dendritic cell obtains a tumor neoantigen (**A**) and travels to a lymph node where it presents it to a naïve T cell (**B**). When the MHC class I molecule containing the tumor neoantigen binds to the TCR, a positive/activating signal is sent. This is called priming. When CD28 on the T cell binds to B7 on the dendritic cell, a positive/activating signal is sent. This is called co-stimulation. Together, these two steps can activate the T cell. T cells also express CTLA-4, which if they bind to B7 can activate an immune checkpoint and down-regulate T cell activation. Anti-CTLA-4 antibodies can abrogate this negative signaling. The activated T cells then enter circulation (**C**). When a T cell arrives at the tumor bed, it engages with a cancer cell that presents antigens (**D**). When it encounters the tumor neoantigen to which it has been activated against, it can mount an immune response (**E**). If PD-L1 or PD-L2 on the cancer cell engages with PD-1 on the T cell, an immune checkpoint is activated, and the T cell receives an inhibitory signal. Anti-PD-1 and anti-PD-L1 antibodies can abrogate this negative signaling. Provided the balance favours activation, the cytotoxic T cell kills the cancer cell (**E**). ++ stimulating signal. −− inhibitory signal. CD28-—cluster of differentiation 28. CTLA-4—cytotoxic T-lymphocyte-associated antigen-4. MHC—major histocompatibility complex. PD-1—programmed death-1. PD-L1/2TCT—programmed death ligand 1/2. TCR—T cell receptor.

Two steps are required for T cell activation: presentation of an antigen to the T cell receptor (TCR) via the major histocompatibility complex (MHC) on the T cell, and a co-stimulatory signal, namely the interaction between CD28 on the T cell and B7 proteins on the antigen presenting cell (APC) [23] (Figure 1). T cells also express CTLA-4 on their surface, which competes with CD28 for binding to B7 proteins on APCs [23]. When CTLA-4 engagement by APCs predominates over CD28, an inhibitory signal is sent and T cell activation is attenuated [24]. Furthermore, activated T cells express PD-1 on their surfaces. When bound by PD-L1 from tumor or other cells, activated T cell functions are reduced and T cell apoptosis is promoted [24]. This also leads to reduced apoptosis of T-regulatory cells (Tregs), further facilitating downregulation of the immune response [24] (Figure 1).

If a cancer cell presents a tumor neoantigen to a T cell, an immune response can be mounted to destroy the cancer cell. However, cancer cells have adapted to exploit these physiologic immunosuppressive mechanisms by upregulating PD-L1 on their cell surface for example, and in doing so, attenuate the anti-tumor immune response. As described in the introduction, ICIs including anti-CTLA-4, anti-PD-1 and anti-PD-L1 antibodies have been developed to restore the anti-tumor immune response (Table 1).

## 3. Mechanisms of ICI Resistance

**Figure 2:**
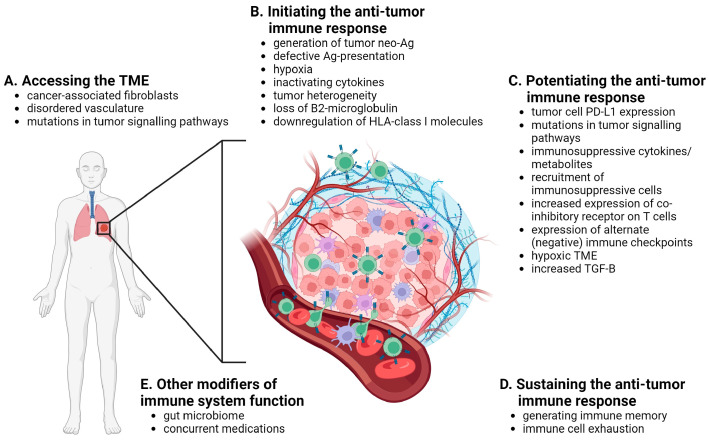
Mechanisms of resistance to anti-PD-1, anti-PD-L1 and anti-CTLA-4 immune checkpoint inhibitors including accessing the TME (**A**), initiating the anti-tumor immune response (**B**), potentiating the anti-tumor immune response (**C**), sustaining the anti-tumor immune response (**D**) and other modifiers of immune system function (**E**). Ag—antigen. B—beta. HLA—human leukocyte antigen. PD-L1—programmed death-ligand 1. TGF—transforming growth factor. TME—tumor microenvironment.

There are different ways to classify mechanisms of resistance to ICIs including temporally (primary vs. secondary), spatially (tumor intrinsic vs. tumor extrinsic) and immunologically (immune-inflamed vs. immune-desert vs. immune-excluded, or based on the sequential stages of immune activation). Here we organize the discussion based on the sequential stages of immune activation: access of immune cells to the tumor microenvironment (TME), initiation of the anti-tumor immune response, potentiating the downstream activation of the effector function of T cells, and sustaining the anti-tumor immune response, as well as a consideration of host factors that influence these stages (Figure 2).

### 3.1. Accessing the TME

Tumor extrinsic factors are largely responsible for impeding immune cells from accessing the TME. In order for cancer cells to respond to ICIs, immune cells must first make it into the TME. Cancer-associated fibroblasts, which reside within the TME, promote tumorigenesis by initiating remodeling of the extra-cellular matrix or by secreting certain cyto- and chemokines [25]. By secreting chemokines, cancer-associated fibroblasts can limit the attraction of effector T cells to the TME, and by remodeling the TME into dense stroma, create a barrier that effector T cells are unable to penetrate [25]. Chemokines (ex. CCL2 and IL-8) have also been affiliated with the recruitment of myeloid-derived suppressor cells (MDSCs) to the TME, which act to further promote an immune suppressive/tumor supportive environment [26].

In addition to these barriers, the disordered vasculature in the TME, characterized by defective endothelial cells, contributes to the exclusion of immune cells [27]. Typically, cells travelling through the bloodstream arrive at their proper destination by adhesion molecules expressed on their cell surface and that of endothelial cells [28]. A lack of adhesion molecules on endothelial cells impedes diapedesis of lymphocytes through the disordered blood vessels of the TME. In keeping with these findings, Hugo et al. reported a gene signature in tumors that exhibited primary PD-1 blockade resistance, which included expression of a set of immunosuppressive cytokines, epithelial-to-mesenchymal transition (EMT) transcription factors and pro-angiogenic factors [29].

Finally, in addition to alterations in pathways related to the TME, activation of other oncogenic pathways within tumor cells (via loss of tumor suppressor genes or activation of proto-oncogenes) have been reported. For example, loss of PTEN expression, alterations in the β-catenin/WNT signaling pathway and concomitant loss of STK11 with activation of KRAS have been associated with a lack of infiltration of T cells to the TME and markers of T-cell exhaustion [30,31,32,33,34].

### 3.2. Initiating the Anti-Tumor Immune Response

Once inside the TME, T cells must be activated against cancer cell-specific/tumor neoantigens (unique epitopes from mutant proteins in cancer cells). The generation of tumor neoantigens is tissue-type specific and those cancers that have higher rates of somatic mutations including lung and melanoma, or those with microsatellite instability such as certain colorectal cancers, have the highest response rates to ICIs [35,36]. Therefore, tumor intrinsic mechanisms including a lack of expression of neoantigens, or defective antigen processing/presentation by tumor cells, as well as tumor extrinsic mechanisms including secretion of inactivating cytokines and creation of a hypoxic environment, may inhibit the response to ICIs by preventing T cell activation. Additionally, due to selective pressures, clones that lack a certain tumor neoantigen targeted by the initial anti-tumor immune response may develop and expand, leading to a clone that evades the anti-tumor immune response and exhibits secondary resistance to ICIs [37].

(Neo)antigens are presented by MHC class I and class II molecules to CD8+ and CD4+ T cells, respectively [38]. MHC class I molecules are present on all nucleated cells and consist of a heavy chain and β2-microglobulin [38]. Chaperone proteins stabilize the MHC class I in the endoplasmic reticulum until it binds to an endogenous peptide (ex. viral peptide, or degraded endogenous “self” protein) and travels to the cell surface where it presents an 8–16 amino acid long peptide [38,39]. Alterations in genes that encode proteins that are a part of this process are known to occur in tumor cells. For example, loss of β2-microglobulin expression or downregulation of human leukocyte antigen (HLA) class I molecules leads to impaired expression of MHC class I molecules on the cell surface [40,41,42]. Downregulation or loss of expression can occur via epigenetic modifications of the genes that encode these proteins.

### 3.3. Potentiating the Anti-Tumor Immune Response

Once primed by binding to a tumor neoantigen, T cells must activate downstream pathways that potentiate an anti-tumor immune response, as described above. This can be subverted by tumor-intrinsic factors including expression of high levels of PD-L1 on tumor cells and mutations of key proteins in pathways that mediate PD-1 blockade resistance (ex. loss of JAK1/2 and interferon-γ (IFN-γ) receptor expression), or tumor-extrinsic factors including promoting expression of immunosuppressive cytokines or metabolites, recruitment of immunosuppressive cells (ex. Tregs, MDSCs, M2-polarised tumor-associated macrophages (TAMs) and T helper 2 (Th2) CD4+ T cells), increased expression of co-inhibitory receptors on T cells themselves (ex. CTLA-4) or expression of alternate immune checkpoint receptors (ex. TIM-3, LAG3, TIGIT) [43,44,45,46].

For example, in the hypoxic TME, MDSCs express hypoxia inducible factor 1α (HIF1α) which upregulates cell surface expression of PD-L1 in both MDSCs and tumor cells. Hypoxia-induced HIF1α expression in tumor-infiltrating lymphocytes (TILs) increases PD-1 expression as well [47]. With increased expression and binding of PD-1 with PD-L1, tumor cells are able to activate the immune checkpoint, promote T cell exhaustion and thereby ICI resistance. As well, high lactate and low glucose levels in the hypoxic TME also leads to gene expression changes in CD4+ and CD8+ effector T cells, including inactivation of nuclear factor of activated T cells (NFATs), which in turn leads to suppression of the production of pro-inflammatory IFN-γ needed to potentiate the anti-tumor immune response [47,48].

As well, tumor cells secrete immunosuppressive cytokines such as transforming growth factor-β (TGF-β) and interleukin-2 (IL-2) which promote the generation of Treg cells instead of effector T cells [49,50]. Differentiation of CD4+ naïve cells into T helper (Th) cells is key to promoting secretion of pro-inflammatory cytokines, which facilitate antibody production and the response to pathogens. TGF-β prevents the acquisition of Th effector functions [51]. Normally, CD8+ naïve T cells readily differentiate into effector cells upon antigen stimulation. The presence of TGF-β potently inhibits CD8+ T cell differentiation and these cells fail to acquire cytotoxic functions. Moreover, TGF-β promotes the differentiation of CD4+CD25- T cells into Tregs, which further prevents immune activation and anti-tumor immunity in response to a presented tumor neoantigen [51,52].

### 3.4. Sustaining the Anti-Tumor Immune Response

Following successful activation of the anti-tumor immune response, it is thought that memory immune cells (ex. effector memory T cells) are responsible for the durable clinical benefit seen in some patients that respond [53,54]. In other patients, T cell exhaustion from chronic inflammation and antigenic stimulation leads to resistance to ICIs. Transcriptomes associated with acute effector, memory and exhausted T cells have been elucidated and evidence suggests that the transcriptional landscape associated with T cell exhaustion is distinct from that of effector or memory CD8+ T cells [55,56]. As such, in patients where T cell exhaustion occurs, the ability of memory CD8+ T cells to sustain an anti-tumor immune response over the long term may be limited [55].

### 3.5. Other Modifiers of the Response to Immune Checkpoint Inhibitors

There are other modifiers of response to ICIs that are more indirect. For example, a patient’s gut microbiome appears to greatly influence functioning of their immune system [57]. While a normal gut microbiome balances tolerance to commensal bacteria and food antigens with defense against pathogenic bacteria, dysbiosis of the microbiome can lead to disruption of the intestinal mucosa within the host, allowing leakage of microbes and metabolites that may lead to a chronic inflammatory state, deregulated cell growth, impair myeloid cell functions (ex. clearing of mutated cells) and overall reduce the patient’s ability to mount an anti-tumor immune response [58].

One mechanism by which an intact host microbiome is thought to contribute to an anti-tumor immune response is through antigen cross-reactivity [59,60]. Cross-reactive CD4+ or CD8+ T cells primed against a bacterial antigen may be able to generate an anti-tumor immune response in cells with the same antigen, in addition to neoantigens. As well, gut microbiota can lead to induction of local immunomodulatory cytokines that disseminate systemically with the capability of shifting the subsets of activated immune cells within the TME towards promoting an anti-tumor immune response [58]. Finally, metabolites secreted by a normal host microbiome may enter circulation and enter the TME, influencing activation of Th cells, antibody secretion and macrophage and dendritic cell function [61,62]. A dysfunctional microbiome can shift these proposed mechanisms towards one that negatively regulates the anti-tumor immune response and renders the patient less responsive to ICIs.

Agents that disrupt a patient’s normal gut microbiome, therefore, can contribute to resistance to ICIs. Concomitant medications including proton pump inhibitors (PPIs) and antibiotics are thought to contribute to gut microbiota dysbiosis due to their ability to alter the balance between non-pathogenic/commensal and pathogenic microbes within the gut [63,64]. Interestingly, the timing of antibiotic administration may be relevant: antibiotics administered after exposure to ICIs may not affect the response and subsequent outcomes like progression-free and overall survival as the normal gut microbiome was unaltered at the time of ICI exposure [65]. In fact, one study demonstrated that exposure to antibiotics after an initial response to ICIs does not affect patient outcomes [65]. Steroids are another commonly prescribed medication to oncology patients that has been demonstrated to impact response and outcomes [66,67]. Interestingly, the reason for prescribing the steroid appears to be indicative of the impact of the steroid on ICI efficacy. For example, steroids (ex. prednisone, dexamethasone) prescribed to manage side effects of treatment or to address immune-related adverse events (irAEs) are thought not to impact efficacy, while steroids prescribed to aid with symptoms like appetite or energy, are associated with a lack of ICI efficacy [67]. While intriguing, this may simply reflect a poorer performance status or tumor biology in a patient who would have had a poor response to ICIs whether taking steroids or not. Interestingly though, a retrospective single institution cohort study showed that in metastatic NSCLC patients, early use of steroids (within 28 days of ICI start) was associated with a poorer disease control rate, progression-free survival (PFS) and overall survival (OS) [68]. Other medications that may affect ICI response include, but are not limited to, selective serotonin reuptake inhibitors (SSRIs), beta blockers, cannabinoids and metformin [69,70,71,72]. The mechanisms by which they are thought to do so are via direct or indirect effects on the host immune system [69,72,73].

## 4. Strategies to Overcome Immune Checkpoint Inhibitor Resistance

There are many therapeutic strategies in development to overcome ICI resistance. These will be elaborated upon in the sections below.

### 4.1. Enhancing T Cell Priming/Tumor Immunogenicity

One strategy to enhance T cell priming/tumor immunogenicity is to increase the amount of tumor neoantigens available to T cells. One way to do this is to combine immunotherapy with other standard therapies such as chemotherapy and radiation therapy. Chemotherapy can act as a double-edged sword. It can disrupt the intestinal mucosa and kill commensal bacteria leading to dysbiosis of the host microbiome [74]. It can also lead to infections that are treated with antibiotics which in turn affect the gut microbiome and can also kill effector T cells in the TME. However, chemotherapy can kill immunosuppressive cells within the TME including MDSCs and Tregs and upon tumor cell killing, can increase the availability and variety of tumor neoantigens that can be presented to T cells that otherwise would not have been seen by the immune system [75,76]. Evidence for its benefit can be found in KEYNOTE-407 and KEYNOTE-189 where NSCLC patients treated with pembrolizumab + chemotherapy had better response rates, PFS and OS versus patients treated with chemotherapy alone [77,78].

Radiation therapy is a localized treatment that can both suppress the immune system, while also leading to an immunogenic cell death [79]. A phenomenon called the Abscopal effect supports the idea that radiation therapy delivered locally can lead to a more systemic anti-tumor immune response as is seen when non-irradiated metastases shrink in response to radiation therapy delivered elsewhere [79]. It is thought that combining radiation therapy with ICIs may potentiate the Abscopal effect [79]. As an example, in a prospective trial of 22 patients with stage IV melanoma, Hiniker et al. report clinical benefit in 50% of patients who received 4 cycles of ipilimumab concurrent with palliative radiation therapy including stable disease, partial and complete responses [80].

Other treatments that lead to immunogenic cell deaths and release of tumor neoantigens that may overcome resistance to ICIs include radiofrequency ablation, cryoablation, transarterial chemoembolization (TACE) and radioembolisation. For example, TACE has been shown to induce an immunogenic cell death with tumor-specific immune responses that can be boosted by anti-CTLA-4 treatment [81].

Switching gears, treatment with oncolytic viruses, which activate innate immunity, elicit an anti-tumor immune response through several mechanisms including increased tumor neoantigen release [82]. The only FDA-approved oncolytic virus, talimogene laherparepvec, is approved for the treatment of unresectable metastatic stage IIIB/C-IVM1a melanoma [83]. It is a modified oncolytic herpes virus which when injected intra-tumorally produces granulocyte-macrophage colony stimulating factor, enhancing the anti-tumor immune response, and tumor cell lysis. While it has shown efficacy locally where injected and occasionally distantly, it lacks responses as a single agent in patients with visceral metastases. Therefore, it is being studied in combination with ICIs including pembrolizumab with promising phase I results [84]. Long term follow-up from a phase 2 trial in stage IIIB-IVM1a melanoma patients with one or more injectable cutaneous, subcutaneous or nodal lesions randomized to neoadjuvant talimogene laherparepvec injection, followed by surgery to surgery alone, demonstrated that injection with the oncolytic virus improved cancer-related outcomes including 5-year recurrence-free survival, event-free survival, distant metastasis-free survival and overall survival. The 5-year overall survival rates were 77.3% vs. 62.7% (HR 0.54; 80% CI 0.36–0.81). The improved survival outcomes are thought to be the results of an induced systemic immunologic anti-tumor effect as there was elevated CD8+ density after treatment with the oncolytic virus [85,86].

Anti-cancer vaccines are also being developed as a strategy to prime tumor-specific T cell activation. The key is to develop a vaccine that is limited to tumor cells so as to reduce toxicity to normal cells [87]. The utility of a cancer vaccine relies on the ability of the host immune system to mount an immune response, which may not occur in the context of immune cell exhaustion and other immunosuppressive mechanisms present in the TME [85]. As such, they have not yet met with much success, including in NSCLC [88,89,90,91,92]. However, the ATALANTE-1 trial in advanced NSCLC patients who progressed following sequential or concurrent chemo and ICI, demonstrated an OS benefit in patients with secondary ICI resistance when treated with OSE2101 versus standard of care next-line chemotherapy (docetaxel or pemetrexed) with a median OS of 11.1 vs. 7.5 months favouring the OSE2101 anti-cancer vaccine (HR 0.59, *p* = 0.017) [93]. OSE2101 is a T-specific immunotherapy designed to induce cytotoxic T lymphocytes against the following tumor-associated antigens: HER2, CEA, MAGE2, MAGE3 and p53 [93]. Because of relatively low success as single agents, anti-cancer vaccines are now being studied in combination with ICIs, chemo- and radiation therapy [87,94,95]. More recently, personalized cancer vaccines based on patient tumor RNA and mRNA (peptide) have demonstrated safety and feasibility with signs of early efficacy, including in NSCLC [96,97,98,99].

Finally, chimeric antigen receptor T cell (CAR-T) therapy, a type of cellular therapy, utilizes gene transfection to express an antigen receptor against a target unique to cancer cells in T cells that are then expanded and administered to patients [100]. While CAR-T therapy has been very successful in certain hematologic malignancies, it has lacked the same success in solid tumors. One reason is that an immunosuppressive TME can affect the activation and subsequent activity of CAR-T cells; pairing with ICIs may help to overcome this roadblock [101]. A second reason is that a remodeled TME designed to exclude immune cells from entering the environment may prevent CAR-T cells from reaching their solid tumor targets [101]. A third is that solid tumors exhibit more tumor heterogeneity and therefore identifying a tumor-associated antigen common to all tumor cells from various clones is difficult [101]. In NSCLC, several targets for CARs are being studied including EGFR, HER2, CEA, MSLN, PSCA, MUC1, ROR1 and PD-L1 [101].

### 4.2. Improving the Immunosuppressive Microenvironment

As mentioned above, the cellular, metabolite and chemo-/cytokine milieu within the TME plays a large roll in response or resistance to ICIs. Indolamine 2,3-dioxygenase 1 (IDO1) is an intracellular enzyme produced by tumor cells, MDSCs and TAMs that metabolizes tryptophan, negatively impacts effector T cell function and enhances Treg activity, thereby inhibiting the anti-tumor immune response [102]. Overexpression of IDO1 in tumor cells has been associated with a poor prognosis [103]. It is thought that inhibition of IDO1 may synergize with ICIs. Accordingly epacadostat, an IDO1 inhibitor, has shown early efficacy and safety in phase I and II trials and is currently being studied in a phase III trial in combination with pembrolizumab in advanced NSCLC patients with a PD-L1 TPS score ≥ 50%, though it’s combination with pembrolizumab in patients with unresectable or metastatic melanoma did not improve PFS or OS over placebo + pembrolizumab [104,105].

Vascular endothelial growth factor (VEGF) plays a role in modifying both the TME and the immune system. VEGF inhibits the maturation of dendritic cells and supports the presence and function of immunosuppressive cells including Tregs, TAMs and MDSCs [106]. As a result, anti-angiogenesis therapies have been studied in combination with ICIs for both their negative effect on angiogenesis and their positive effect on the immunosuppressive TME. Combinations of ICIs and various VEGF tyrosine kinase inhibitors (TKIs) has been met with success clinically most notably in renal cell carcinoma and more recently in NSCLC [107,108,109,110]. The IMpower 150 trial demonstrated an improved PFS and OS with atezolizumab (A; anti-PD-L1 antibody) + bevacizumab (B; anti-VEGF-A antibody) + carboplatin (C) + paclitaxel (P) (ABCP) compared to BCP (median PFS 8.3 vs. 6.8 mos, HR 0.62, *p* < 0.001 and median OS 19.2 vs. 14.7 mos, HR 0.78, *p* = 0.02 both favouring ABCP) in the first line for advanced NSCLC patients without driver mutations [111]. As well, a phase I trial showed early efficacy of ramucirumab (anti-VEGFR-2 antibody) + pembrolizumab in both previously treated and untreated advanced NSCLC [112,113]. More recently, the S1800A substudy of the phase II Lung-MAP trial demonstrated a significantly increased median OS with ramucirumab + pembrolizumab versus standard of care chemotherapy in advanced NSCLC patients previously treated with ICI and platinum-based chemotherapy who experience secondary resistance to ICI (median OS 14.5 vs. 11.6 months, HR 0.69, *p* = 0.05) [114].

Another important signaling molecule in the TME is TGF-β, which is involved in Treg activation and angiogenesis [49]. In a murine model of BRAF V600E/PTEN null melanoma transgenic mice, an oral TGF-β inhibitor combined with intraperitoneal anti-CTLA-4 antibody led to an increased CD8+ effector T cell: Treg cell ratio and suppression of primary and metastatic melanoma tumor growth [115]. The inhibition of other immunosuppressive chemokines including CXCL12 and CXCR4 are also under investigation [116,117,118].

Activated macrophages can be M1 polarized (fostering an inflammatory response against pathogens/tumor cells) or M2 polarized (exerting an immunosuppressive phenotype which favours tissue repair but also tumor progression) [102,119]. The colony-stimulating factor 1 (CSF-1) cytokine in the TME acts to maintain M2 polarization of macrophages and proliferation of TAMs and its receptor has been shown to be upregulated de novo or in response to ICIs [120,121]. Efforts to reprogram TAMs towards M1 polarization with an anti-CSF-1 receptor antibody are in early development. When used in combination with single agent ICI or gemcitabine, murine models of pancreatic ductal adenocarcinoma have exhibited efficacy [122].

Because MDSCs serve to downregulate the response to ICIs (ex. melanoma patients with higher numbers of MDSCs have a poorer response to ipilimumab), attempts to impair MDSC function to enhance ICI efficacy are underway. Entinostat, a histone deacetylase (HDAC) inhibitor, has been shown to negatively affect MDSCs [123,124]. Recently, the ENCORE-601 phase II trial reported promising results in melanoma patients treated with entinostat + pembrolizumab who had progressed on previous ICI therapy, though an expansion cohort in NSCLC failed to meet criteria for a significant overall response rate [125,126].

Finally, oncolytic viruses can also be used to enhance T-cell infiltration and reprogram the chemo-/cytokine milieu of the TME via type I interferon induction. It is thought that this may facilitate transforming an inhibitory TME to one that allows an anti-tumor immune response to be mounted. One other advantage of oncolytic viruses is that they can be engineered to encode proteins including ICIs, bi-specific T cell engager molecules, costimulatory receptor ligands and chemo-/cytokines that attract effector T cells to the TME. These proteins are produced as the virus replicates within tumor cells, augmenting anti-tumor immunity. One caveat to their use is a lack of persistence in the TME, as the anti-tumor immune response they generate serves to kill the cells they infect and replicate in. Strategies to address this are underway and many oncolytic viruses are being investigated in clinical trials as monotherapies, or in combination with chemotherapies, ICIs, tumor vaccines and radiation therapy.

### 4.3. Reversing T Cell Exhaustion

The idea of T cell exhaustion was first coined in 1993 by Moskophidis who described ineffective cytotoxic abilities of a murine model exposed to chronic viral infection, marked by the removal of antigen-specific T cells [127]. This exhaustion has also been documented in humans with chronic viral infections. While it refers mostly to defective cytotoxicity (CD8+ T cells), CD4+ T cells have also been observed to become ineffective during chronic infections [128]. Exhausted T cells lose their effector function, proliferative capacity, granzyme B and perforin expression, limiting their cytotoxic abilities [129]. Together with other metabolic and replication changes, these lead to the over-expression of co-inhibitory receptors on the surface of T cells, and T cell apoptosis [129].

As outlined above, anti-PD-1 antibodies prevent PD-1-mediated attenuation of the T cell receptor downstream activation cascade as a result of binding to a presented antigen and prevents T cell apoptosis. As well, anti-CTLA-4 antibodies lead to a reduction in Tregs within the TME and have been shown to modulate the T cell receptor repertoire. Both effects are proposed to reinvigorate T cells, or reverse exhaustion; however, they are not the whole story. Due to the presence of alternative checkpoints, T cell exhaustion can still occur, or remain, even when exposed to ICIs [129].

T cell immunoglobulin and mucin-domain containing-3 (TIM-3) is a co-inhibitory receptor expressed on IFN-γ-producing T cells, certain Treg cells and other innate immune cells [130]. Activation of the TIM-3 receptor leads to inhibition of Th1 cells and the expression of pro-inflammatory tumor necrosis factor (TNF) and IFN-γ cytokines, as well as cytotoxic T cell function. High levels of TIM-3 expression are associated with T cell exhaustion [130]. Combinations of ICIs and anti-TIM-3 antibodies are currently being studied. A phase I trial of the TIM-3 inhibitor cobolimab as monotherapy or in combination with anti-PD-1 antibodies nivolumab or dostarlimab have shown early safety and efficacy in patients with advanced solid tumors (predominantly NSCLC and melanoma) previously treated with multiple lines of therapy [131]. Sabatolimab, another anti-TIM-3 antibody, has also been studied alone or in combination with an anti-PD-1 antibody (spartalizumab) in patients with advanced solid tumors in a phase I/Ib study [132]. Sabatolimab was ineffective as monotherapy but showed safety and preliminary efficacy when combined with spartalizumab [132].

Lymphocyte-activation gene 3 (LAG3) has been shown to inhibit T cell effector function through its interaction with MHC class II molecules, though other ligands with less well-known functions have been described [133]. Its expression is a hallmark of exhausted CD4+ and CD8+ T cells and co-expression with PD-1 in T cells is associated with resistance to ICIs. Many therapies targeting LAG3 are under investigation including monoclonal antibodies and bispecific drugs. Recently, a phase III trial investigating relatlimab (an anti-LAG3 antibody) in combination with nivolimab in untreated advanced melanoma patients reported safety and efficacy. At 12 months, the PFS rate for the combination was 47.7% vs. 36.0% with nivolumab alone, and median PFS was 10.1 months vs. 4.6 months (HR 0.75, 95%CI 0.62–0.92; *p* = 0.006) [134]. At a median follow-up time of 19.3 months, the median OS was not reached for the combination, and 34.1 months for nivolumab alone (HR 0.80, 95%CI 0.64–1.01; *p* = 0.059) [135]. Bispecific LAG3 immunotherapies target LAG3 and PD-1 or PD-L1. One such example is FS118, a first-in-class human tetravalent, full-length human IgG1 anti-LAG3/PD-L1 bispecific antibody [133]. In vitro it has been shown to overcome PD-L1- and LAG3-mediated inhibition of T cell activation and effector function [133]. In a phase I first-in-human study of patients with advanced solid tumors who failed previous anti-PD-1/PD-L1 therapy, FS118 did not result in dose-/treatment-limiting toxicities [136]. Interestingly, disease stabilization for >6 months was observed in patients who had previously developed secondary anti-PD-1/-PD-L1 resistance (defined as initial complete response, partial response or stable disease ≥ 3 months with previous anti-PD-1/-PD-L1 therapy), but not in patients who had exhibited primary resistance [136].

Finally, another important alternative immune checkpoint receptor is T cell immunoreceptor with immunoglobulin and ITIM domain (TIGIT). TIGIT is expressed on NK cells, CD4+, CD8+ T cells and Treg cells and binds to two ligands (CD155 and CD112) that are expressed by tumor cells and other APCs within the TME [137]. Binding its ligands induces inhibition of T and NK cells, and cells in the TME like dendritic cells to secrete immunosuppressive cytokines (ex. IL-10) and decrease production of pro-inflammatory cytokines (ex. IL-12) [136]. In Treg cells, TIGIT signaling enhances their immunosuppressive functions and interestingly, Fap2 protein from gut microbiota binds to TIGIT and triggers other inhibitory signals on T and NK cells in the TME [138]. One anti-TIGIT antibody, tiragolumab, showed promising activity in NSCLC in a randomized phase II placebo-controlled trial of tiragolumab with atezolizumab vs. atezolizumab alone in the first line treatment of EGFR/ALK wildtype NSCLC patients who were PD-L1 TPS positive [139]. At an average of 30.4 months of follow-up, the combination therapy had a better overall response rate (38.8% vs. 20.6%), median PFS (5.6 vs. 3.9 months; HR 0.62, 95%CI 0.42–0.91) and median OS (23.2 vs. 14.5 months; HR 0.69, 95%CI 0.44–1.07) versus atezolizumab alone, though OS did not reach significance [140]. The results appeared to be driven by the subgroup of patients that had a PD-L1 TPS score ≥ 50% where ORR (69% vs. 24%), median PFS (16.6 vs. 4.11 months; HR 0.29, 95%CI 0.15–0.53) and median OS (not reached vs. 12.8 months; HR 0.23, 95%CI 0.10–0.53) significantly favoured tiragolumab + atezolizumab over atezolizumab alone [140]. No OS benefit was seen in patients with PD-L1 TPS < 50%. An ongoing phase III study (SKYSCRAPER-01) in advanced NSCLC patients with PD-L1 TPS ≥ 50% failed in meeting its co-primary endpoint of PFS and demonstrated a numerical but not statistically significant benefit in OS of tiragolumab + atezolizumab versus atezolizumab alone based on the disclosure of a second interim analysis [141]. Other clinical trials with anti-TIGIT antibodies combined with immunotherapy are ongoing.

Other co-inhibitory receptors expressed on effector T cells include VISTA, B7-H3 and BTLA and these are currently under clinical investigation [102,142]. In addition to co-inhibitory receptors, efforts to develop agonists of co-stimulatory receptors (ex. CD137, glucocorticoid-induced TNFR related protein (GITR), OX40 and CD27) are also underway [102]. Co-stimulatory receptors increase proliferation and differentiation of effector T cells, enhancing T cell activation. Many compounds with agonist-like effects towards these co-stimulatory receptors are currently under investigation as monotherapy or with other ICIs.

In addition to leveraging alternative immune checkpoint inhibitors, cellular therapy utilizing TILs is showing early promise in addressing T cell exhaustion. TILs are a group of mono-nuclear immune cells that include T and B lymphocytes, macrophages, dendritic cells and NK cells [143]. Together, this group of cells can bind multiple tumor-associated antigens with the potential to overcome the antigen escape associated with the tumor heterogeneity of solid tumors [144]. However, TILs lose this function when they become exhausted. When these cells are extracted from patients and expanded ex vivo, they become reinvigorated [145]. When subsequently transfused into patients it is though they can lead to higher response rates and tumor regression. Early trials in NSCLC in the late 1980’s were unsuccessful due to low response rates and a low magnitude of response [146,147]. More recently, Ratto et al. studied TILs in the adjuvant setting for resected stage II/III NSCLC patients. While there was no benefit for stage II and little benefit for stage IIIa patients, stage IIIb patients had a significantly better OS rate at 3 years (*p* < 0.01) [148]. As this trial took place prior to 1996, it is not directly relevant to patients today as adjuvant treatment has changed greatly in the last decade [149,150,151,152]. More recent trials are studying TILs in the metastatic setting. One phase I study of patients who progressed on first line therapy but remained ICI naïve demonstrated safety and early efficacy with autologous TIL + IL-2 treatment including 6 patients with radiographic response, two of which were complete responses [145]. Another group studied single agent LN-145 TILs (without IL-2) in a phase II study of patients who had previously received 1–3 lines of prior therapy. All patients had previously received ICI. The overall response rate was 25%, including one complete response and there was stable disease in another 50% of patients [153]. One important consideration for this cellular therapy, however, is the need to remove and expand patient TILs which can take 6 to 8 weeks. Many NSCLC patients would not have the time to wait as disease may progress and affect their performance status to a degree that precludes treatment. As well, infused T cells have a short half-life, which may prevent them from reaching the solid tumor site [143]. Finally, infusion with TILs is preceded by non-myeloablative chemotherapy and followed by IL-2 infusions. The associated anticipated toxicities of this treatment, which include bone marrow suppression, hypoxia, hypotension, diarrhea and fevers may exclude patients without adequate cardiopulmonary reserve from treatment [143].

### 4.4. Combining ICIs with Small Molecule Inhibitors

In addition to chemotherapy and radiation therapy as mentioned above, small molecule inhibitors have been studied in combination with ICIs to overcome ICI resistance across a variety of the mechanisms listed above. The most common of which are tyrosine kinase inhibitors (TKIs) that include lenvatinib, axitinib, sunitinib and cabozantinib for example, in NSCLC, RCC and endometrial cancers, although other small molecules like cobimetinib (MEK inhibitor) and PARP inhibitors like olaparib and niraparib are also being studied in colorectal, ovarian and prostate cancers [108,109,110,154,155,156,157,158,159,160,161,162].

Cabozantinib is a TKI that targets multiple tyrosine kinase receptors (TKRs) including AXL, MET and VEGFR [163]. In addition to the role for VEGF in modulating the tumor microenvironment and immune system, AXL also regulates the anti-tumor immune response. Activation of AXL leads to immune evasion by up-regulation of BCL-2 and Twist, suppression of Toll-like receptor (TLR) inflammatory signaling, suppression of NK cells, and limiting the expression of pro-inflammatory cytokines [164,165]. AXL has also been shown to suppress antigen presentation via MHC-I and enhance the expression of cytokine and chemokines that result in a limited up front immune response [166]. AXL has been implicated in chemo-resistance as well as ICI resistance. A recent study demonstrated that high AXL transcript levels in tumors from melanoma patients were significantly correlated with resistance to anti-PD-1 ICIs [29]. Therefore, combining ICIs with TKIs such as cabozantinib have the potential to overcome ICI resistance. This may have been the case in the CheckMate 9ER trial comparing nivolumab + cabozantinib to sunitinib alone (the then standard of care first line treatment) in treatment-naïve clear cell RCC patients [108]. The trial met its primary endpoint where patients treated with combination therapy had a PFS of 16.6 months vs. 8.3 months (HR 0.51; 95%CI 0.41–0.64; *p* < 0.001) and the OS rate at 12 months was 85.7% vs. 75.6% in favour of the nivolumab + cabozantinib. The ORR also favoured combination therapy: 55.7% vs. 27.1% with sunitinib alone [108]. Longer term follow-up (median 32.9 months) demonstrated a median OS of 37.7 months vs. 34.3 months in favour of nivolumab + cabozantinib (HR 0.70; 95%CI 0.55–0.90; *p* = 0.0043) [167]. This strategy was not as successful in non-small cell lung cancer. Two recently presented studies have failed to show a significant benefit of adding TKIs that inhibit AXL to ICIs. The CONTACT-01 trial failed to show an improvement in OS when comparing cabozantinib plus atezolizumab to docetaxel (mOS of 10.7 vs. 10.5) in NSCLC patients who had progressed on prior ICI and platinum doublet [168]. In a similar design, the SAPPHIRE clinical trial evaluated sitravatinib and nivolumab versus docetaxel [169]. There was a modest improvement in overall survival that was not statistically significant (mOS 12.2 vs. 10.6) [169]. Further work in development of a biomarker to select the optimal patients could be beneficial. Other AXL inhibitors continue to be studied in combination with ICIs [165].

Other small molecule inhibitors like PARPi, are thought to overcome ICI resistance by enhancing the number of tumor neoantigens and the TMB of tumors. In cell lines and animal models, the combination of PARPi with PD-L1 blockade resensitized PARPi-treated cells to T-cell killing [170,171]. Additionally, BRCA2 has been shown to be significantly more frequently mutated in tumors that respond to anti-PD-1 therapy, suggesting that exacerbating DNA damage and genomic instability may improve response to ICIs [29]. Unfortunately, their combination has not been met with much clinical success. The phase 3 KEYLYNK-010 study looking at pembrolizumab + olaparib in patients with metastatic castration-resistant prostate cancer who progressed following chemotherapy and either abiraterone or enzalutamide was discontinued for futility [172]. KEYLYNK-001, a phase III study looking at chemotherapy +/− concurrent and maintenance pembrolizumab followed by maintenance with olaparib or placebo in BRCA1/2 non-mutated epithelial ovarian cancer tumors and KEYLYNK-008, a phase III study evaluating maintenance olaparib and pembrolizumab have completed recruitment and results are awaited [173,174].

Finally, Imblaze370 looked at atezolizumab +/− cobimetinib (MEKi) versus regorafenib in previously treated metastatic colorectal cancer patients in the third line setting [159]. Preclinical models combining MEK inhibitors with ICI showed additional activity over the individual drugs alone, and in mouse models, treatment with cobimetinib alone impeded tumor growth, and in combination with anti-PD-L1 therapy, tumor growth was further inhibited and some complete responses were observed [175,176,177]. A phase 1b-2 trial showed promising results of atezolizumab + cobimetinib in metastatic colorectal cancer patients, leading to the rationale for this phase III trial [159,178]. Patients were stratified by, but not selected by, Ras status (wild type vs. mutant). Unfortunately, the primary endpoint of improved OS of the two experimental arms versus standard of care regorafenib was not met [159]. Subgroup analysis did not reveal a trend toward clinical efficacy between the three treatment groups for patients with RAS mutation or high PD-L1 expression. The authors posit that the combination was insufficient to overcome the non-immunogenic phenotype of microsatellite stable metastatic colorectal cancer [159].

### 4.5. Addressing Other Modifiers of Response to ICIs

Reversing dysbiosis and restoring a patient’s normal/healthy gut microbiome has met with success as a strategy to improve response to ICIs in primary or acquired resistance settings [58,59,179]. There are several strategies to positively influence a patient’s gut microbiota including administering probiotics, prebiotics or through fecal microbial transplant (FMT). Probiotics, more specifically bacterial consortia, contain selective live bacteria (ex. Lactobacillus or bifidobacteria) and have been shown to improve outcomes in cancer patients when treated with ICIs by altering the level of important pro-inflammatory cytokines including IFN-γ and TNF in the TME [58,180]. The first prospective study of delivery of a live bacterium was conducted in metastatic renal cell carcinoma patients receiving dual ICI therapy with nivolumab and ipilimumab [181]. Thirty patients were randomized to receive the probiotic + dual ICI or dual ICI alone. Of the evaluable patients, those that received the probiotic demonstrated increased presence of the main constituent bacterium *C. butyricum*, fewer pathogenic species (ex. *E. coli* and *Klebsiella* spp.), a significantly higher response rate (59% vs. 11%, *p* = 0.024) and a significantly longer median PFS (not reached vs. 11 weeks, *p* < 0.001) [181]. Studies of other probiotics are underway [58].

Prebiotics (ex. oligofructose, resistance starch or inulin) are fermentable non-digestible substrates that promote the growth of healthier non-pathogenic bacteria and lead to a more diverse and healthier gut microbiome. Depending on the cytokine, metabolite and immune cell milieu of the TME, prebiotic administration can enhance tumor cell killing efficacy by promoting both effector T cell and Treg subsets [58]. Preclinical data suggests that prebiotics may enhance the efficacy of chemotherapy or reduce the side effects of chemo- and radiation therapy and studies of their combination with ICI therapy are underway [182,183,184,185].

Finally, FMT in combination with ICIs has been shown to reprogram the TME to promote an anti-tumor immune response in melanoma, prostate and gastrointestinal cancers [58]. FMT is the most direct way to alter a patient’s gut microbiome. In FMT, stool from a donor is given to a recipient via oral administration of lyophilized or frozen pills, or via scope to the upper or lower intestines with the goal of restoring a healthy gut microbiome that will positively modulate a patient’s immune system to mount an anti-tumor immune response [58]. The combination of FMT with ICIs in treating patients with primary and acquired resistance to ICIs is underway in many clinical trials and has already demonstrated success in phase I trials of melanoma patients with primary and acquired resistance to anti-PD-1 therapy [186]. For example, in one study, 20 previously untreated patients with advanced melanoma were treated with healthy donor FMT and either pembrolizumab or nivolumab [187]. There was an acceptable safety profile with combination therapy. The objective response rate was 65% including four patients with complete responses. All 20 patients had successful engraftment of bacterial strains from the healthy donors however over time, only the patients whose microbiomes continued to increase in similarity to the healthy donor microbiome responded to therapy. As well, only responders saw a decrease in the presence of deleterious bacteria.

As previously mentioned, concurrent medications that modulate the immune system such as antibiotics, PPIs and steroids, may affect ICI efficacy. While these may be easily modifiable (ex. switching a patient from a PPI to an H2-blocker for reflux), there is insufficient evidence to make recommendations to do so.

## 5. Conclusions and Future Directions

There are many ways in which patients present with primary or acquired resistance to ICIs. Efforts to reverse resistance include ways to combat T cell exhaustion, reprogram the TME into one favourable for promoting an anti-tumor immune response, increase the availability of tumor neoantigens, target alternative immune checkpoints, restore a normal/healthy patient gut microbiome, oncolytic viruses and tumor vaccines. The most studied and/or most promising methods include combining ICIs with chemotherapy, radiation therapy and other treatments that lead to immunogenic cell deaths, therapies targeting alternative immune checkpoints or other pathways involved in immunomodulation such as with small molecule inhibitors and restoring a normal/healthy patient gut microbiome. Future efforts to improve the efficacy of all manner of immunotherapies will include improving our ability to predict responses with appropriate biomarkers in order to pre-select patients for effective therapies, and to continue to investigate mechanisms of primary and acquired resistance that may be targeted. We remain in our infancy with respect to understanding the complex interplay between cancer and the immune system, but the progress we have seen has brought more than glimmers of hope to cancer patients and researchers alike.

## Figures and Tables

**Table 1:** List of immune checkpoint inhibitors and their targets that are being (clinicaltrials.gov, accessed on 19 September 2023), or have previously been, tested in phase III clinical trials.

Drug Name	Target
Cemiplimab	PD-1
Dostarlimab	PD-1
Nivolumab	PD-1
Pembrolizumab	PD-1
Retifanlimab	PD-1
Spartalizumab	PD-1
Tislelizumab	PD-1
Zimberelimab	PD-1
Atezolizumab	PD-L1
Avelumab	PD-L1
Durvalumab	PD-L1
Ipilimumab	CTLA-4
Tremelimumab	CTLA-4
Fianlimab	LAG3
Relatlimab	LAG3
Cobolimab	TIM-3
Sabatolimab	TIM-3
Domvanalimab	TIGIT
Ociperlimab	TIGIT
Tiragolumab	TIGIT
Vibostolimab	TIGIT
Enoblituzumab	B7-H3
Epacadostat	IDO
Cadonilimab	Bispecific- PD-1 and CTLA-4
Tebotelimab	Bispecific- PD-1 and LAG3
Feladilimab	Costimulatory ICOS receptor agonist
Evorpacept	CD47 inhibitor fused to inactive IgG Fc
Utomilumab	Phae IB/III trial CD-137 agonist
Eftilagimod alpha	Solubilized LAG3 and MHC class II agonist
